# Mucosal-Associated Invariant T Cells in Autoimmune Diseases

**DOI:** 10.3389/fimmu.2018.01333

**Published:** 2018-06-11

**Authors:** Asako Chiba, Goh Murayama, Sachiko Miyake

**Affiliations:** Department of Immunology, Juntendo University School of Medicine, Tokyo, Japan

**Keywords:** mucosal-associated invariant T cells, multiple sclerosis, systemic lupus erythematosus, inflammatory arthritis, inflammatory bowel diseases, diabetes, asthma

## Abstract

Mucosal-associated invariant T (MAIT) cells are innate T cells restricted by MHC-related molecule 1 (MR1). MAIT cells express semi-invariant T-cell receptors TRAV1-2-TRAJ33/12/20 in humans and TRAV1-TRAJ33 in mice. MAIT cells recognize vitamin B2 biosynthesis derivatives presented by MR1. Similar to other innate lymphocytes, MAIT cells are also activated by cytokines in the absence of exogenous antigens. MAIT cells have the capacity to produce cytokines, such as IFNγ, TNFα, and IL-17, and cytotoxic proteins, including perforin and granzyme B. MAIT cells were originally named after their preferential location in the mucosal tissue of the gut, but they are also abundant in other peripheral organs, including the liver and lungs. In humans, the frequency of MAIT cells is high in peripheral blood, and these cells constitute approximately 5% of circulating CD3^+^ cells. Their abundance in tissues and rapid activation following stimulation have led to great interest in their function in various types of immune diseases. In this review, first, we will briefly introduce key information of MAIT cell biology required for better understating their roles in immune responses, and then describe how MAIT cells are associated with autoimmune and other immune diseases in humans. Moreover, we will discuss their functions based on information from animal models of autoimmune and immunological diseases.

## Introduction

Two subsets of T cells express semi-invariant T-cell receptors (TCRs). The first subset includes the thoroughly studied invariant natural killer T (iNKT) cells that uniquely recognize lipid antigens presented by CD1d, a homolog of the MHC molecule. TCRα rearrangement in iNKT cells includes Vα24–Jα18 (TRAV10-TRAJ18) in humans and Vα14–Jα18 (TRAV 11–TRAJ 18) in mice. The second subset, mucosal-associated invariant T (MAIT) cells, are restricted by the MHC-related protein 1 (MR1) and express Vα7.2–Jα33 (TRAV1-2–TRAJ33) in humans and Vα19–Jα33 (TRAV1–TRAJ33) in mice (1). Vα7.2–Jα33 rearrangement was discovered by Porcelli et al. along with Vα24–Jα28 during analysis of the TCR repertoire of human CD4^−^CD8^−^ (double-negative; DN) T cells (2). Later, Tilloy et al. discovered homologous Vα19–Jα33 in mice (1). MAIT cells were originally named after their preferential location in the gut lamina propria. Their absence in germ-free mice also indicated their association with mucosal immunity (3). In 2009, Martin et al. generated a monoclonal antibody against human Vα7.2 TCR and demonstrated that Vα7.2TCR^+^ cells with high expression of CD161 were MAIT cells (4). Human MAIT cells are abundant in peripheral blood and constitute up to 10% of blood CD3^+^ cells. Because the frequency of iNKT cells in human peripheral blood is 0.01–1%, MAIT cells are 10- to 1,000-fold more frequent than iNKT cells.

Recent studies using MR1 tetramers revealed that not all of the TCR usage of human MAIT cells is restricted to TRAV1-2–TRAJ33 (5). Approximately, 30% of MR1-restricted TRAV1-2^+^ cells use TRAV1-2 joined with TRAJ20 or TRAJ12 gene segments (5). Moreover, subsets of MR1-resticted T cells do not express TRAV1-2, and their features are discussed elsewhere (6). TRAV1-2–TRAJ33 are mostly paired with TRBV6-6 and TRBV20 (1, 5, 7). In mice, only TRAV1–TRAJ33 (Vα19–Jα33) has been reported as a murine MAIT TCR paired with TRBV13-3 (Vβ8.1), TRBV 13-2 (Vβ8.2), and TRBV19 (Vβ6) (1, 5, 8). The usage of different MAIT TCRs might be related to the tissue distribution of MAIT cells. Vα7.2–Jα33 is the dominant MAIT TCR Vα transcript in human peripheral blood, but the percentages of Vα7.2–Jα12 transcripts are higher than those of Vα7.2–Jα33 transcripts in kidney and intestine biopsies from some individuals (7).

## Mait Cell Phenotype

Mucosal-associated invariant T cells in adult blood exhibit the effector memory phenotype (CD95^hi^CD62L^lo^CD45RO^+^CD45RA^lo^ CD27^+^ CD122^+^) (4, 9). In the thymus and cord blood, MAIT cells display a naïve phenotype and are present at very low numbers (4). However, these MAIT cells already express CD161 in the thymus and CD161 and IL-18Rα in cord blood (10) and produce TNFα in response to *Mycobacterium tuberculosis*-infected cells (11). MAIT cells also express PLZF, a master regulator of innate-like T cells (8, 12). PLZF expression appears important in the development of MAIT cells because PLZF deficient mice have a significantly lower frequency of MAIT cells. Approximately 95% of human MAIT cells are DN or CD8^+^. Most MHC-restricted CD8^+^T cells express the CD8αβ heterodimer, but CD8^+^ MAIT cells express CD8αα homodimers, and some of them coexpress the CD8αβ heterodimer (4, 5, 13). Approximately 60% of MAIT cells were CD4^−^CD8^−^ in most tissues of C57BL6/J mice except for lymph nodes where 40% of MAIT cells were CD4^+^. CD8^+^ MAIT cells were more frequent in Balb/c mice than in C57Bl/6J mice, but CD4^+^MAIT cells were also enriched and constituted half of MAIT cells in lymph nodes (8).

Human peripheral blood MAIT cells are CCR5^+^CCR6^+^CCR7^−^CCR9^+/−^ CXCR3^−^CXCR4^+/−^ CXCR6^+^ (10, 14, 15). Mouse MAIT cells are CCR6^+/−^ CCR9^+/−^ CXCR6^hi^ but negative for CCR4, CCR7, CXCR1, CXCR3, and CXCR4 (8). Lack of CCR7 and CD62L expression indicates their poor ability to migrate into lymph nodes *via* high endothelial venules, and expression of CCR9 and CXCR6 suggests their ability to migrate into the intestine and the liver. In fact, human MAIT cells are abundant in peripheral blood and enriched in tissues such as the liver (20–50% of CD3^+^ cells), intestine (1–10% of CD3^+^ cells), and lung (2–4% of CD3^+^ cells) (5, 10, 16–21). Human MAIT cells are also detected in other tissues, including female genital mucosa, kidney, prostate, and ovary (7, 22). FTY720, an agonist of sphingosine-1-phosphate receptors, inhibits the egress of naïve and central memory T and B cells from lymph nodes. FTY720 has been used for treatment of patients with multiple sclerosis (MS). FTY720 treatment decreased the total lymphocyte count but increased MAIT cell frequency; it also reduced DN cells and increased CD8^hi^ and CD4^+^cells among MAIT cells (23). This finding indicates that MAIT cells are indeed rare in lymph nodes, and tissue distribution may differ among subsets of MAIT cells. Activated MAIT cells may obtain more migrating capacity because IL-18-stimulated MAIT cells express very late antigen-4 (VLA-4), an integrin important for migration into the site of inflammation (24). No antibody against murine Vα19TCR is available, and the frequency of MAIT cells in mice was unknown until the recent development of MR1 tetramers (8). Compared with iNKT cells, MAIT cells are relatively rare in laboratory strains of mice except for CAST/EiJ mice (1, 3, 25). The average frequency of MAIT cells among C57BL/6 mouse lymphocytes is 3.3, 0.7, 0.6, 0.2, 0.08, and 0.05% in the lung, lamina propria, liver, lymph nodes, spleen, and thymus, respectively (8).

## Mait Cell Activation Mechanisms

Early studies demonstrated that MAIT cells are deficient in germ-free mice and activated by antigen-presenting cells in the presence of bacteria in an MR1-dependent manner (3, 26, 27). These findings suggested that MAIT cells might recognize microbial antigens presented by the MR1 molecule. Microbes that activated MAIT cells included various types of bacterial species and yeast. In 2012, Kjer-Nielsen et al. described several MR1-restricted antigens. They identified 6-formylpterin (6-FP), a photodegradation product of folic acid (vitamin B9), as an MR1 ligand. 6-FP upregulated surface expression of MR1 but failed to activate MAIT cells. The researchers found that reduced 6-hydroxymethyl-8-d-ribityllumazine (rRL-6-CH2OH) derived from the bacterial riboflavin (vitamin B2) biosynthetic pathway is a MAIT cell-activating MR1 ligand (28). Later, Corbett et al. revealed that some potent MR1 ligands, including 5- (2-oxopropylideneamino)-6-d-ribitylaminouracil (5-OP-RU), are produced by an interaction between early intermediates in the bacterial riboflavin synthesis pathway and either glyoxal or methylglyoxal, and these antigens are unstable unless they are captured and stabilized by the MR1 molecule (29). More recently, several MR1 ligands have been reported among drugs and drug metabolites, such as diclofenac and methotrexate (30). A photodegraded product of aminopterin or methotrexate captured by the MR1 molecule inhibited MAIT cell activation by 5-OP-RU, whereas diclofenac and its metabolites stimulated MAIT cells.

**Figure 1:**
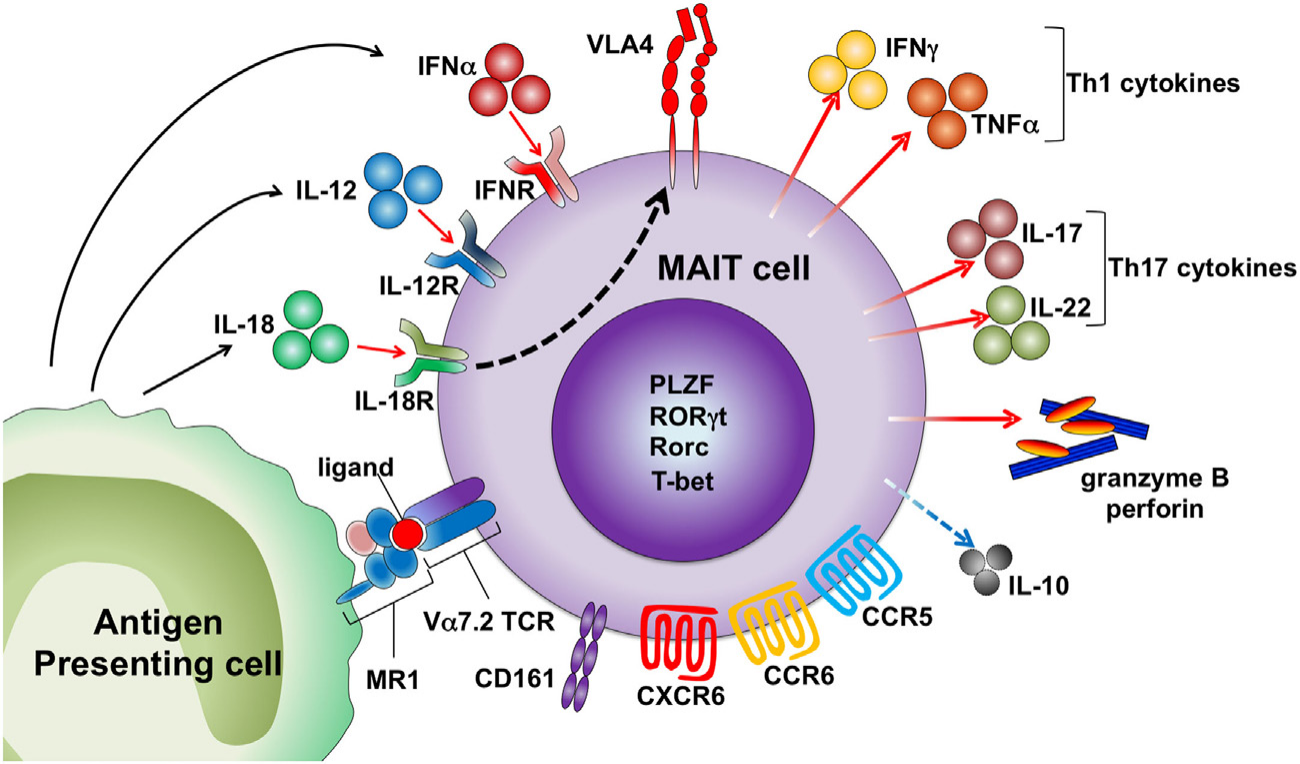
Activation mechanisms of human mucosal-associated invariant T (MAIT) cells. MAIT cells are activated by the MR1 ligand derived from microbes and drugs. Cytokines, including IL-12, IL-18, and IFNα, also activate MAIT cells by an antigen-independent mechanism. MAIT cells express several chemokine and cytokine receptors and homing receptors. Activated MAIT cells upregulate very late antigen-4 (VLA-4) and secrete granzyme B and perforin, Th1/Th17 cytokines, and low levels of IL-10.

Similar to iNKT cells, MAIT cells are activated by cytokines in an MR1-independent manner (Figure 1). MR1 expression is indispensable for the development of MAIT cells but not for the effector functions of these cells. Our group demonstrated that MAIT cells exacerbated joint inflammation in arthritis models, and MAIT cells exerted their effector function even when they were adoptively transferred into MR1-deficient mice (31). A MAIT cell-enriched population from V19iTCR transgenic (Vα19iTg) mice produced IL-17 after exposure to IL-23 and proliferated upon IL-1β stimulation (31). Inhibition of bacterial growth of *Mycobacterium* by MAIT cells was more dependent on IL-12-mediated activation of these cells rather than on MR1 antigen recognition by MAIT cells (32). Human MAIT cells express high levels of IL-18Rα and are activated to produce IFNγ by IL-12 plus IL-18 (33–37). MAIT cells are also activated by type I IFN (33, 34). The kinetics of MAIT cell activation upon different types of stimuli might differ as activation of MAIT cells at early time points after incubation with *E. coli* was MR1-dependent, and IL-12 + IL-18-mediated activation took more time (35). MAIT cells are activated by TCR signals (anti-CD3/CD28) when they are stimulated in the presence of other peripheral blood mononuclear cells, but sorted MAIT cells (CD4^−^CD8^+^CD56^+^CD16^−^CD161^hi^Vα7.2^+^ cells) did not respond to TCR signals. However, sorted MAIT cells produced IFNγ and granzyme B when they were activated with TCR signals in the presence of pro-inflammatory signals provided by monocytes activated by TLR agonists. Sorted MAIT cells also produced cytokines, such as IFNγ and TNFα, in response to IL-12/15/18 stimulation (37). Because MAIT cells are enriched in mucosal tissue where MR1 antigens produced by commensal bacteria are present, they may be programed to respond only when they are exposed to such antigens together with inflammatory signals to avoid unwanted tissue inflammation.

## Mait Cell Function

Upon stimulation with phorbol 12-myristate13-acetate (PMA) and ionomycin or anti-CD3 and anti-CD28, human MAIT cells produce IFNγ, TNFα, IL-17, IL-2, and granzyme B (10, 14, 26). Mouse spleen MAIT cells produce high levels of IL-17 and MIP-1α and low levels of IL-10, IFNγ, and TNFα following stimulation with PMA and ionomycin or anti-CD3 and anti-CD28 (8). This bias toward IL-17 production was also observed in MAIT cells in various types of organs, such as the thymus and lung (8). Unsurprisingly, mouse MAIT cells express high levels of retinoic acid-related orphan receptor (RORγτ) and low levels of T-bet (8). Human MAIT cells also express Rorc and T-bet; as expected, the expression of T-bet is higher than that of Rorc (10, 38). MAIT cells in different tissues may vary in their cytokine-producing capacity. Mouse thymus MAIT cells also produce other cytokines, such as GM-CSF, IL-4, and IL-13 (8). Mouse MAIT cells from iVα19–Vβ6 transgenic mouse spleen produced IL-2 after stimulation by *E. coli*-infected dendritic cells (26). In humans, adipose tissue MAIT cells but not peripheral blood MAIT cells produce more IL-10 than IL-17. Interestingly, MAIT cells in adipose tissue from obese individuals produced more IL-17 and less IL-10 (39).

## Factors Affecting Mait Cell Frequency and Function

Mucosal-associated invariant T cell numbers are very low in peripheral blood at birth, and their frequency increases with age up to 40–50 years of age (9, 10, 40). Novak et al. studied MAIT cells from individuals at different ages and demonstrated that the frequency of MAIT cells is highest in women of fertile age and significantly declines in elderly individuals (40). MAIT cells are decreased in patients with type 2 diabetes (T2D) and/or obesity (39, 41, 42). Upon stimulation with PMA and ionomycin, MAIT cells from T2D patients produced higher levels of IL-17, and MAIT cells from obese T2D patients produced even higher levels of other cytokines, including IL-2, granzyme B, and IFNγ. In obese patients, MAIT cell frequency was higher in omental adipose tissue than in peripheral blood; moreover, MAIT cell frequency was increased, and cytokine-producing ability was decreased after bariatric surgery. Smoking appears to reduce the frequency of peripheral blood MAIT cells (43). Circulating MAIT cells are affected by not only systemic but also inhaled administration of corticosteroids (43). Some drugs and drug metabolites have been reported as MR1 ligands (30). MR1 ligands derived from microorganisms should be abundantly present in the gut. Although whether the frequency and function of MAIT cells are affected by drugs or indigenous microbes remains unknown, taking the possible influence of these factors into account when studying human MAIT cells might be important.

## Mait Cells in Autoimmune and Immunological Diseases

**Figure 2:**
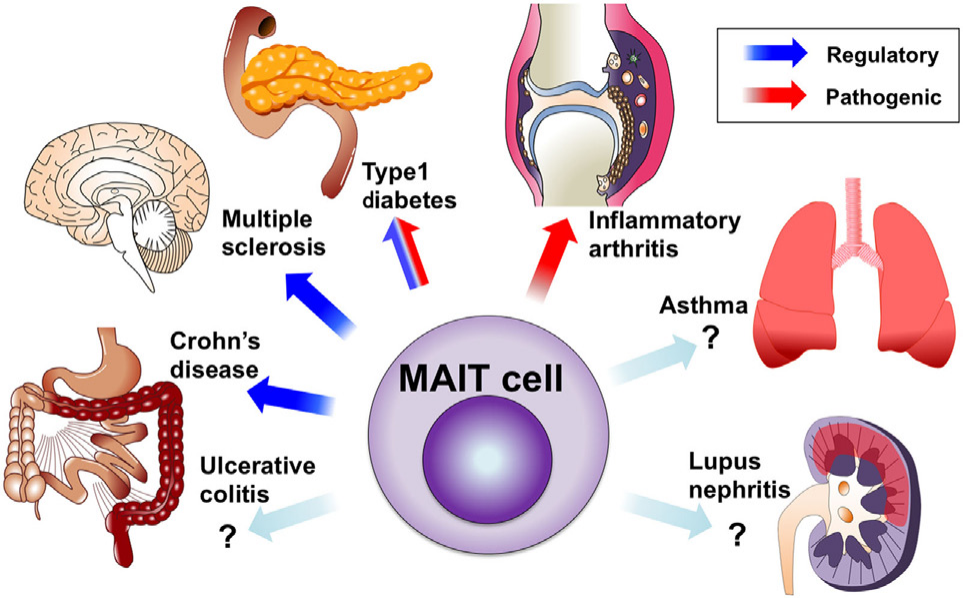
Mucosal-associated invariant T (MAIT) cells in autoimmune and immunological diseases. Circulating MAIT cells are reduced in various autoimmune and immunological diseases. MAIT cells are accumulated or present in inflamed tissues, including the central nervous system, intestine, lungs, and joints. The protective roles of MAIT cells were demonstrated in animal models of multiple sclerosis and Crohn’s disease, as well as onset of type 1 diabetes (T1D). The possible contribution of MAIT cells to tissue inflammation was demonstrated in arthritis models and pancreatitis in T1D.

The role of MAIT cells in immunological disorders was largely unknown until our group described their protective role against experimental autoimmune myelitis (EAE), an animal model of MS, and their pro-inflammatory roles in arthritis models. Since anti-Vα7.2 TCR monoclonal antibody has become available, many groups, including ours, have conducted studies on MAIT cells in autoimmune and immunological diseases. MAIT cells appear to be involved in various types of diseases (Figure 2), but their contribution to each pathology is currently unknown. This has been mostly due to the lack of good tools to study murine MAIT cells. MAIT cells are usually rare in mice and there is no monoclonal antibody against murine MAIT cell TCR. New technical approaches such as MR1 tetramers and CAST/EiJ mice may overcome these issues. Here, we review MAIT cells in human immunological diseases and the corresponding animal models. In human studies, MAIT cells are identified by the Vα7.2 TCR and high CD161 expression unless otherwise specified.

### Multiple Sclerosis

Multiple sclerosis is an inflammatory demyelinating disease affecting the central nervous system (CNS). Although the etiology of MS is not fully understood, MS is considered an autoimmune disease against the myelin component of the CNS. Illes et al. investigated invariant TCR expression in the CNS lesions of patients with MS by using the single-strand conformation polymorphism clonotype method (44). They found very low Vα24–Jα18 expression in MS CNS samples but observed Vα7.2–Jα33 expression in half of MS CNS samples and in most cerebral spinal fluid (CSF) samples. Later, the presence of Vα7.2TCR^+^CD161^+^ in MS lesions was confirmed by other groups (24, 45, 46). CD8^+^MAIT cells are present in MS brain lesions, and approximately 5% of CD8^+^T cells were Vα7.2TCR^+^CD161^+^ cells in acute and chronic active MS lesions, suggesting infiltration of MAIT cells into MS lesions (24). These studies were performed by using MS autopsies, and a more recent study demonstrated Vα7.2–Jα33 transcripts in the brain lesions of a MS patient with newly onset disease (47). There are several conflicting reports regarding the frequency of circulating MAIT cells in MS patients. Most reports demonstrate the reduction of all or subsets of MAIT cells in MS except for one report showing an increase in CD161^hi^ CD8^+^T cells, and most of these cells are usually MAIT cells (48). Two reports demonstrated the reduction of V7.2TCR^+^ CD161^high^ cells or CD161^high^ memory CD8^+^T cells in MS (14, 24). Other groups showed that the frequency of MAIT cells was comparable between MS patients and healthy volunteers (23, 46), but MAIT cells were reduced in patients with progressive disease (46), and CD8^hi^ cells among MAIT cells were decreased in MS (23). IFNβ treatment did not affect the frequency of MAIT cells in MS patients (23), but the frequency of MAIT cells in patients with relapse was increased along with clinical recovery after steroid treatment. Several findings suggest migration of MAIT cells into the CNS. *In vitro*, IL-18 increased surface expression of VLA-4 on CD8^+^ MAIT cells, and the frequency of CD8^+^MAIT cells was inversely correlated with the serum level of IL-18 in MS patients but not in healthy individuals (24). MS MAIT cells overexpress P-selectin glycoprotein ligand-1 (PSGL-1) and CD11a (part of the lymphocyte function-associated antigen 1), which are important for cell rolling and homing though the blood–brain barrier (46).

Therefore, what role do MAIT cells play in the pathogenesis of MS? IFNγ and TNFα production by MAIT cells was decreased in untreated MS patients, but FTY720 treatment recovered the cytokine-producing capacity of MAIT cells (23). Successful treatment of MS with autologous hematopoietic stem cell transplantation was accompanied by depletion of CD8^+^MAIT cells, whereas regulatory T cells and CD56^high^ natural killer cells were increased in the peripheral blood of MS patients (45). These findings indicated a pro-inflammatory role for MAIT cells in MS, whereas MAIT cells played a protective role in EAE, an animal model of MS (49). The disease development and progression were suppressed in Vα19iTg mice, and MR1-deficient mice developed more severe EAE than did control mice. Adoptive transfer of T cells enriched with a MAIT cell population protected wild-type mice from EAE. Inhibition of EAE in Vα19iTg mice was associated with decreased Th1 and Th17 responses against myelin oligodendrocyte glycoprotein and increased secretion of IL-10. Cytokines produced by Vα19iT cells are different from those produced by human MAIT cells. IL-10 is heavily produced by Vα19iT cells, but human MAIT cells produce very little IL-10. Whether MAIT cells play a protective role in the development of human MS is unknown. As depletion of MAIT cells (CD5^+^CD19^−^ TCRγδ^−^ CD161^high^Vα7.2TCR^+^ cells) increased IFNγ production by T cells *in vitro*, human MAIT cells may also have suppressive effect on other T cells (14).

### Systemic Lupus Erythematosus (SLE)

Systemic lupus erythematosus is a systemic autoimmune disease that affects various types of organs, including the skin, kidneys, and CNS. The most characteristic features of SLE are the production of autoantibodies targeting nucleic acids and immune activation by the generation of nucleic acid-containing immune complexes. Thus, the impaired tolerance of T and B cells has been considered one of the major causes of the disease. However, abnormalities in function and number have been reported in innate lymphocytes including natural killer cells and iNKT cells in patients with SLE (50–56). The frequency of MAIT cells was also reduced in the peripheral blood of SLE patients, and the reduction of these cells was more profound than that of γδT cells and iNKT cells (33). We confirmed that the reduction of MAIT cells in SLE was not a result of downregulation of surface markers by single-cell PCR for the expression of Vα7.2–Jα33 TCR. Moreover, the reduction of MAIT cells was not due to the use of corticosteroids in SLE. Lupus MAIT cells were less responsive to stimuli and prone to death, and there were more apoptotic cells among circulating MAIT cells in SLE. Cho et al. also reported the decrease of circulating MAIT cell in SLE patients (57). They showed impaired IFNγ production by lupus MAIT cells that was accompanied by elevated PD-1 expression and an intrinsic defect in the Ca2^+^/calcineurin/NFAT1 signaling pathway of these cells. In our study, MAIT cells from SLE patients with active disease expressed high levels of CD69, and the activated status of MAIT cells positively correlated with disease activity. Thus, MAIT cells in SLE patients appear to be activated and lost due to activation-induced cell death *in vivo*; moreover, the remaining MAIT cells are less responsive to stimuli. We elucidated two possible mechanisms of MAIT cell activation. First, monocytes from SLE patients exerted higher MR1 antigen-presenting capacity to MAIT cells. Second, elevated IFNα appeared to be associated with activation of MAIT cells in SLE. Overexpression of type I IFNs and IFN-inducible genes has been reported in SLE patients, and type I IFN is thought to play a central role in the pathogenesis of lupus (58). CD69 expression on MAIT cells positively correlated with serum levels of IL-18 and IFNα in SLE, and exposure to IFNα-induced MAIT cell activation *in vitro*, suggesting that these cytokines may also contribute to the activation of MAIT cells in SLE. MAIT cells migrate into inflamed tissues including kidneys (59). MAIT cells constitutively express chemokine receptors, and exposure to IL-18 upregulates the surface expression of VLA-4 (24), which mediates T-cell migration through an interaction with vascular cell adhesion molecule (VCAM-1). Urinary levels of IL-18 and VCAM-1 were increased and associated with nephritis activity in SLE (60, 61). DN T cells infiltrated the kidneys in lupus, and the majority of these cells were neither γδT cells nor iNKT cells (62). Thus, MAIT cells may migrate into inflamed tissues in SLE.

### Inflammatory Arthritis

Rheumatoid arthritis (RA) is the most common inflammatory arthritis that typically affects the small joints of the hands and feet, and the synovium is the primary site of inflammation. RA is characterized by production of rheumatoid factor (RF) and anti-citrullinated protein antibody. Spondyloarthritis (SpA) is a group of disorders comprising ankylosing spondylitis (AS), psoriatic arthritis, reactive arthritis, arthropathy of inflammatory bowel disease (IBD), and undifferentiated SpA. The features of SpA include the absence of RF and association with *HLA-B27*; the main targets are the enthesis and axial skeleton. Neutralizing antibodies against the TNFα and IL-6 signaling pathways are widely used in RA treatment; in addition to TNF inhibitors, blocking the IL-23/IL-17 axis is beneficial in AS. Circulating MAIT cells are reduced in patients with RA and SpA including AS (63–65). MAIT cells displayed enhanced IL-17-producing capacity and activated status of these cells correlated with disease activity in AS (63, 64). Cell death of circulating MAIT cells was not enhanced, but they were accumulated in the synovial fluid (SF) in AS. SF MAIT cells displayed high levels of CD69 and enhanced producing capacity of IL-17 and granzyme B in AS. Additionally, SF MAIT cells are enriched in RA (57). Interestingly, IL-17 production by SF MAIT cells was higher in AS than in RA, but TNFα- and IFNγ-producing SF MAIT cells in AS were comparable to those in RA (64). IL-7R polymorphisms are associated with AS, and IL-7 primes MAIT cells (20, 66). Gracey et al. demonstrated that IL-7R expression is increased on AS MAIT cells, and exposure to IL-7 exacerbated the IL-17-producing capacity of AS MAIT cells. Considering their capacity to produce inflammatory cytokines at the site of tissue inflammation, MAIT cells appear to contribute to tissue inflammation in arthritis. In animal models of inflammatory arthritis, MAIT cells enhanced arthritic inflammation (31). DBA1J mice immunized with type II collagen (CII) develop collagen-induced arthritis (CIA), and MR1 deficiency attenuated the disease severity of CIA. Because MR1 deficiency had little effect on T and B cell responses against CII, MAIT cells appeared to contribute to the effector phase of arthritis. In fact, MAIT cell deficiency reduced the disease severity of collagen antibody-induced arthritis (CAIA), and adoptive transfer of a T-cell population enriched with iVα19 TCR^+^ cells from iVα19 TCR T g mice enhanced CAIA in MR1-deficient mice. This iVα19 TCR^+^ cell population was activated by IL-1β or IL-23 in the absence of exogenous antigens. Therefore, MAIT cell activation in the CAIA model may be mediated by cytokines and does not require TCR stimulation.

### Inflammatory Bowel Diseases

Inflammatory bowel diseases are chronic relapsing disorders of the gastrointestinal tract, comprising Crohn’s disease (CD) and ulcerative colitis (UC). CD affects the distal ileum and colon, and UC involves only the colon. Inflammation in UC is superficial and includes the mucosa and submucosa, whereas CD involves transmural inflammation. The etiology of IBD is not fully understood, but the clinical efficacy of neutralizing antibodies specific for TNFα indicates the role of cytokine-producing immune cells in IBD (67). Both innate and adaptive immune systems appear to contribute to the pathogenesis of IBD. CD is thought to be mediated by Th1 and Th17 cell responses against gut commensal microbiota. UC is believed to be mediated by Th2 responses; however, anti-IL-13 therapy was not beneficial, and cytokines involved in the pathogenesis of UC appear to be more complicated (68, 69). MAIT cells are reduced in the peripheral blood of patients with CD and UC (18, 70–72). However, IL-17 production by MAIT cells was increased in UC patients (71). CD69 expression on MAIT cells was associated with disease activity. Increased IL-17 production by MAIT cells and correlation of CD69 expression on these cells with disease activity indicated the association of MAIT cells with the pathogenesis of UC. T cells expressing CD161, IL-23R and RORγt are enriched in intestinal mucosa from patients with IBD; thus, MAIT cells may be associated with tissue inflammation in IBD (67, 73, 74). In fact, MAIT cells accumulated in the inflamed mucosa of patients with CD and UC (18, 71, 72). Plasma IL-18 levels were positively correlated with CD69 expression on MAIT cells in UC. Thus, the reduction of circulating MAIT cells may be a result of the recruitment of these cells to the inflamed tissue. A report by Hiejima et al. showed reduced MAIT cell frequency in intestinal mucosa from IBD patients (70). They demonstrated enhanced cell death of MAIT cells in peripheral blood in IBD patients and inflamed mucosa of those with CD. Further studies are required to understand the differences among reports and their function in IBD pathology. One report demonstrated that adoptive transfer of Jα33^+^ cells into mice reduced the severity of intestine inflammation in 2,4,6-trinitrobenzene sulfonic acid (TNBS) colitis, suggesting their protective role in an animal model of CD (75).

### Type 1 Diabetes (T1D)

Harms et al. investigated CD161^bright^ CD8^+^ T cells in patients with juvenile T1D whose clinical onset was within 12 months and demonstrated there was no reduction of these cells in the peripheral blood of patients (76). The frequency of CD27^−^MAIT cells was increased in patients, and these cells displayed more enhanced IL-17-producing capacity. CD161^bright^ CD8^+^T cells increased with age in control individuals but not in juvenile T1D patients, suggesting that circulating MAIT cells may be decreased in patients with long-standing T1D. More recently, a lower frequency of MAIT cells was detected in children with recent onset T1D than in control children (77). MAIT cells from T1D patients expressed increased levels of CD25 and PD-1 and displayed enhanced cytokine production, including TNFα and granzyme B. Because human MAIT cells exerted cytotoxic activity against a pancreatic β-cell line, MAIT cells may contribute to β-cell destruction in T1D.

In non-obese diabetic (NOD) mice, the frequency and number of MAIT cells was lower in the spleen and pancreatic lymph nodes of NOD mice than in those of C57BL/6 mice. MAIT cells were present in pancreatic islets and enriched in the ileum of NOD mice, and these MAIT cells exhibited a more activated phenotype. The recruitment of MAIT cells into the ileum decreased and that into the pancreas increased with aging. IFNγ and granzyme B production by islet MAIT cells was already observed in prediabetic mice and further increased in diabetic mice. These findings indicate that MAIT cells are recruited to the pancreas and contribute to tissue inflammation. However, MR1 deficiency increased the rates of diabetes in NOD mice and the streptozotocin-induced T1D model (77). MR1 deficiency increased intestinal permeability, and this issue was associated with increased infiltration of lymphoid cells into the lamina propria and more bacterial translocation from the gut to pancreatic lymph nodes. Thus, MAIT cells appear to be important for the maintenance of tissue integrity, but they contribute to tissue damage once inflammation occurs.

### Asthma

Asthma encompasses chronic airway inflammation characterized by increased airway hypersensitivity to various types of antigen-specific and non-specific stimuli. Th2 cytokines, such as IL-4, IL-5, and IL-13, play important roles in activating other cells including eosinophils. MAIT cell frequency was reduced in patients with asthma in peripheral blood, sputum, and endobronchial biopsy specimens (78, 79). The reduction in MAIT cell frequency was associated with disease severity, inhaled corticosteroid dose, respiratory function, and disease duration. Moreover, CD69^+^MAIT cells were associated with respiratory function (80). MAIT cells are present in the lung at similar or higher frequencies than those in peripheral blood and are enriched in the lung under inflammatory conditions, including infection (6, 81). Thus, MAIT cells may be involved in the pathogenesis of asthma, but it is difficult to determine the role MAIT cells play in asthma because human MAIT cells mostly produce Th1 and Th17 type cytokines. Therefore, studies of MAIT cells at the site of inflammation or using animal models are required to understand their role in asthma.

## Concluding Remarks

Mucosal-associated invariant T cells appear to be involved in various types of immune disorders, and circulating MAIT cells were reduced in most diseases. We speculate that these findings are due to their unique characteristics. MAIT cells are very sensitive to stimuli, can be activated by antigens and cytokines, and have the capacity to migrate to inflamed tissues. There are several conflicting findings regarding MAIT cell frequencies in some diseases among different research groups. This discrepancy may be due to the methodology used to identify MAIT cells. Other potential reasons include the influence of factors, such as age, gender, obesity, and smoking, on MAIT cells (43). MR1 ligands derived from commensal microbes or drugs could modify MAIT cell function or frequency. Thus, these factors might have influenced these different findings. Studies using new tools such as MR1 tetramers and MR1 ligands may answer these questions and determine the potential of MAIT cells as a therapeutic target in immune diseases.

## Author Contributions

AC, GM, and SM wrote the manuscript. Both AC and SM contributed equally to this work. GM drew schematics.

## Conflict of Interest Statement

The authors declare that the research was conducted in the absence of any commercial relationships that could be construed as a potential conflict of interest.
